# Healthcare systems’ readiness to address gender-based violence during COVID-19 and beyond in Pakistan: a mixed methods study

**DOI:** 10.1186/s12978-026-02356-3

**Published:** 2026-08-19

**Authors:** Rozina Karmaliani, Arusa Lakhani, Laila Ladak, Marina Baig, Yasmin Parpio, Salima Somani, Gabriela Garcia Camacho, Armando Seuc, Moazzam Ali

**Affiliations:** 1 The Aga Khan University, Stadium Road, Karachi, Pakistan; 2 World Health Organization, Geneva, Switzerland

**Keywords:** Gender-based violence, COVID-19, Sexual and reproductive health, Health system

## Abstract

**Background:**

The COVID-19 pandemic has exacerbated gender-based physical, financial, sexual, and psychological harm on a global scale, resulting in a surge of gender-based violence incidents. Consequently, there has been an increased demand for Sexual Reproductive Health Services (SRHS) in response to the pandemic. This study aims to comprehend the impact of COVID-19 on contraception, comprehensive abortion care, sexually transmitted infections (STIs) prevention and treatment, and Gender-based violence (GBV) support at both sub-national and facility levels.

**Methodology:**

An explanatory sequential mixed-methods study was conducted from October 2021 to September 2022 across 9 public and private healthcare facilities in Sindh, Pakistan. Quantitative data were collected from 250 participants (156 women and 94 partners), and facility readiness assessments were conducted using WHO-adapted Service Availability and Readiness Assessment tools. Qualitative in-depth interviews were subsequently conducted with purposively selected women, partners, and 33 healthcare providers until thematic saturation was achieved. Quantitative and qualitative findings were integrated during analysis and interpretation.

**Results:**

Only 33.3% (3/9) of facilities routinely screened for domestic violence, 11.1% (1/9) provided referral linkages to external GBV support services, and 11.1% (1/9) had healthcare providers trained in GBV response within the preceding six months. Health Management Information Systems documented GBV cases in only 11.1% (1/9) of facilities. Qualitative findings indicated increased violence during the pandemic, driven by economic stress, unemployment, and restricted mobility, while sociocultural stigma, lack of trained personnel, and weak referral systems limited service utilization.

**Conclusion:**

The study identified notable deficiencies in healthcare facilities’ preparedness to address instances of violence against women. These deficiencies encompassed the absence of national guidelines, limited resources, inadequate identification methods, scarce referral services, and insufficient privacy measures. Moreover, a lack of training for healthcare practitioners jeopardizes their capacity to provide adequate care and support to GBV victims and survivors.

**Supplementary Information:**

The online version contains supplementary material available at 10.1186/s12978-026-02356-3.

## Plain language summary

Globally, the COVID-19 pandemic engendered suffering across physical, economic, sexual, and psychological dimensions. Notably, distinct instances of gender-based violence (GBV) emerged as nations grappled with containment measures for the pandemic. This study examined the readiness of Pakistani healthcare facilities to address the impact of the COVID-19 pandemic on GBV.

Lockdowns, stressors, and economic upheavals collectively contributed to a surge in gender-based violence during the pandemic. Consequently, victims found themselves trapped with their abusers, exacerbating domestic violence on a global scale. Survivors grappled with public shaming and entrenched patriarchal norms. Additionally, access to sexual and reproductive health services became more challenging due to financial constraints, healthcare provider shortages, and migration restrictions.

The study investigated the preparedness of Pakistani hospitals to respond to GBV. Through baseline and endpoint interviews with pregnant and non-pregnant women, their partners, and healthcare providers, researchers examined nine hospitals in urban and rural settings of Sindh, Pakistan.

Findings revealed healthcare facilities’ inadequacies in addressing gender-based assault, despite a heightened demand for support services. Few facilities provided connections to external support agencies or adhered to national GBV guidelines. Moreover, survivors faced barriers to care due to healthcare personnel’s lack of resources and training in handling GBV cases.

Qualitative analysis indicated that GBV escalated during the pandemic due to familial conflicts and financial strains. Despite an increasing need for assistance, survivors encountered social stigma, a dearth of empathy, and limited resources. To mitigate GBV, the study advocates leveraging technology for counseling, involving law enforcement and religious leaders, and enhancing the availability of support services. In conclusion, addressing GBV preparedness in healthcare facilities requires urgent attention from governments, healthcare professionals, and communities.

**Supplementary Information:**

The online version contains supplementary material available at 10.1186/s12978-026-02356-3.

## Background

COVID-19, designated as a pandemic by the World Health Organization (WHO) in March 2020 [1], swiftly proliferated globally, resulting in widespread physical, financial, sexual, and psychological repercussions for millions of individuals. The WHO’s COVID-19 Situation Report 2020 documented 20,730,456 cases and 751,154 deaths [1], with over 664 million new cases and more than 6.7 million deaths reported by January 25, 2023 [2]. Intimate partner violence prevalence stands at approximately 27% among women aged 15–49, with 13% experiencing such violence in the past year. This issue is notably more prevalent in low and middle-income countries than in high-income ones. For instance, in central Africa, the prevalence of intimate partner violence in the last 12 months was 32%, while it was 24% in eastern sub-Saharan Africa, 19% in South Asia, and 4 to 6% in Europe and North America [3]. Globally, 243 million women and girls aged 15 to 49 have experienced sexual and physical violence by intimate partners in the last 12 months, a trend likely to worsen amid deteriorating economic and health conditions due to the pandemic [4]. Additionally, in Pakistan, the prevalence of intimate partner violence ranges between 13 to 14%, encompassing physical, psychological, and sexual violence [5]. Prior to the COVID-19 pandemic, gender-based violence prevalence in Pakistan was already estimated at approximately 13–14%; however, pandemic-related economic stressors, mobility restrictions, and social isolation were reported to exacerbate both the incidence of violence and barriers to reporting and service utilization [6].

COVID-19’s economic impact has led to a significant surge in Gender-Based Violence (GBV) cases against women worldwide. GBV, as outlined by the United Nations (UN) Commission on the Status of Women, encompasses domestic violence rooted in gender, inflicting physical, sexual, financial, and psychological harm on women, thereby constraining their personal and social freedom [7]. The WHO characterizes GBV as a pattern of coercive behavior aimed at exerting power and control over individuals in intimate relationships through intimidating, threatening, harmful, or harassing actions [8, 9].

The COVID-19 crisis has heightened the demand for Sexual Reproductive Health Services (SRHS). SRH encompasses holistic well-being, physical, emotional, mental, and social, related to all aspects of sexuality and reproduction, emphasizing more than just the absence of disease, dysfunction, or infirmity [10]. The International Conference on Population and Development (ICPD) underscores women’s human rights to freely and responsibly control matters pertaining to their sexuality, devoid of coercion, discrimination, or violence [10, 11]. Consequently, SRH emerges as a fundamental human right, entitling everyone to the highest attainable standard of care for Sexual and Reproductive Health and Rights (SRHR) [12].

Unfortunately, women encountering SRH needs encounter barriers in accessibility, availability, and utilization of SRH services, particularly during the COVID-19 pandemic. The United Nations Population Fund (UNFPA) highlights these challenges, which are compounded by movement restrictions imposed as part of COVID-19 containment measures, reduced healthcare workforce due to infections, inadequate medical resources, and time constraints for SRH patients as healthcare workers grapple with COVID-19-related issues [1].

This paper contributes to the "Health Systems Analysis on Women’s Reproductive and Psychosocial Health Related to Barriers to Availability, Utilization, and Readiness of Sexual and Reproductive Health Services in COVID-19 Affected Areas in Pakistan: A Qualitative and Quantitative Approach." The study endeavors to comprehend how COVID-19 impacts contraception, comprehensive abortion care, STI prevention and treatment, and GBV support at sub-national and facility levels. It aims to help policymakers and health administrators enhance community-responsive policies and services. The overarching goal is to identify crucial gaps and barriers in delivering comprehensive sexual and reproductive health services during health emergencies and offer strategies applicable across various settings to assess health facility and system readiness for future health crises. This study delves into a specific analytical component of the health system associated with GBV during the COVID-19 pandemic.

This study is conceptually guided by the World Health Organization’s Health System Six Building Blocks framework, which examines service delivery, health workforce capacity, health information systems, access to essential medicines and technologies, health financing, and leadership and governance. Applying this framework enabled systematic assessment of healthcare facility readiness to prevent and respond to gender-based violence and provided a structured lens for interpreting system-level barriers identified during the COVID-19 pandemic.

## Methodology

### Study design and timelines

This study employed an explanatory sequential mixed-methods design. Quantitative facility readiness assessments and participant surveys were conducted first to assess the availability, accessibility, and readiness of GBV-related services across participating facilities. Subsequently, qualitative in-depth interviews with women, partners, and healthcare providers were conducted to explain, contextualize, and elaborate on the quantitative findings. Integration of the two datasets occurred at three stages: (1) design, through complementary sampling of participants; (2) analysis, by comparing quantitative readiness indicators with qualitative themes; and (3) interpretation, using joint displays and integrated discussion of findings.

Data collection occurred at two time points: baseline and endpoint, with a 6-month interval between them. Baseline data collection took place between October and December 2021, while endpoint data collection occurred between July and September 2022. The purpose of having two data collection points was to monitor and illustrate changes in services amidst the COVID-19 pandemic and over time.

The health system assessment included a cross-sectional survey of 9 healthcare facilities, a quantitative survey of women and their partners, and qualitative interviews with healthcare providers to capture changes in GBV service availability resulting from the COVID-19 epidemic.

#### Study setting

The study was conducted across nine healthcare facilities in Sindh, Pakistan (LMIC), encompassing rural, semi-urban, urban, primary, secondary, and tertiary care settings.

#### Study participants

The total study population comprised 250 individuals, including 156 women (both pregnant and non-pregnant) and 94 men (partners), who sought SRH services at the healthcare facilities. Additionally, 33 healthcare providers (HCPs), including medical doctors, nurses, nurse assistants, and allied health professionals working at these facilities, were part of the qualitative aspect of the study.

#### Sampling procedure

Health facilities were purposefully selected based on their capacity to provide reproductive health services, care, and support for women experiencing GBV, considering factors such as hospital capacity, administrative rank, rural or urban location, resources, epidemic status, and providers’ willingness to participate. The aim was to ensure a heterogeneous mix of facilities representing various socioeconomic backgrounds and locations.

All women and their partners visiting these nine facilities for reproductive health services during the study period were approached and invited to participate until the target number was reached. Those willing to participate were taken to a separate room, where data was collected after obtaining written consent. After quantitative data collection, participants were invited to participate in qualitative interviews. For in-depth interviews (IDIs), a purposive sampling approach was used, including five to seven women of reproductive age (until saturation), three to five men (until saturation), and two to three HCPs per healthcare facility.

Eligibility criteria for women included being free of physical diseases and aged 18 or older. HCPs stationed in SRH clinics and delivering SRH services for at least six months before the pandemic began were included in the study.

Quantitative participants were recruited through consecutive sampling of eligible clients attending participating facilities during the study period. The sample size was determined based on feasibility across nine facilities and expected service utilization during the pandemic period. Qualitative sampling followed purposive maximum-variation principles, and interviews continued until no new themes emerged across two consecutive interviews, indicating thematic saturation.

#### Data collection and analysis

##### Data collection instrument

The study utilized two quantitative tools to evaluate the provision of sexual and reproductive health services, including GBV services, during the COVID-19 pandemic. The first tool was a health system survey instrument that assessed compliance with and adaptation of WHO’s Service Availability and Readiness Assessment (SARA) and Harmonized Health Facility (HHF) assessment tools, as well as the WHO Health Emergency Department’s health facility checklist for infrastructure and service delivery. The second tool evaluated the readiness and availability of GBV services in healthcare facilities based on five indicators: a) Policies and plans; b) Maintenance and referrals of services; c) Infrastructure; d) Commodities; and e) Human resources. Additionally, an individual participant survey instrument was employed to investigate GBV service utilization and access during the pandemic. Furthermore, in-depth interview guides for women, men, and healthcare providers (HCPs) explored perceptions, experiences, and recommendations for enhancing GBV services in healthcare facilities during the pandemic. These tools underwent validation through expert reviews and pilot testing, with necessary adjustments made before final implementation.

##### Data collection

Health system survey tools were provided to hospital administrations for completion, while an interviewer-administered survey questionnaire was utilized to collect data from individual participants enrolled in the study. Qualitative data were gathered by trained data collectors using open-ended interview guides. Questionnaires were prepared in English and translated into the local language (Urdu). Each in-depth interview (IDI) lasted approximately 40–60 min and was audio-recorded, with researchers also documenting participants’ nonverbal cues. Interviews were conducted in separate rooms at healthcare facilities, with female researchers interviewing women and male researchers interviewing men.

#### Data analysis

Data were entered into SPSS for statistical analysis. Descriptive analysis was employed to examine participants’ basic characteristics and the availability and utilization of GBV services during COVID-19. Audio-recorded data were transcribed verbatim and de-identified, utilizing ID numbers instead of names. Interviews conducted in Urdu were translated into English and back-translated to ensure information integrity. Qualitative coding followed a hybrid inductive-deductive approach using predefined readiness domains while allowing new themes to emerge. Two independent researchers coded transcripts using NVivo software, and discrepancies were resolved through consensus discussions to enhance analytical rigor. Content analysis was employed, involving the condensation of data into codes, the aggregation of similar codes into categories and subcategories, and the synthesis of related categories into themes conveying the data’s meaning.

#### Ethical considerations

Ethical approval was obtained from the WHO Ethics and Research Committee and the local Research Ethics Review Committee (IRB/ERC # 2021–5915-17,041). Informed consent was obtained from all participants after providing detailed explanations of the study’s purpose and procedures. Participants were assured of the voluntary nature of their participation and the absence of negative consequences for refusal. To safeguard participant anonymity, all identifying data were omitted during transcription. Audio files, transcriptions, and electronic data were encrypted and stored in a designated virtual repository. Participants reporting domestic violence during interviews were assisted in accessing relevant support services. Field data collection adhered strictly to COVID-19 safety guidelines.

## Results

The study recruited 250 participants from nine sites in Pakistan. Descriptive statistics summarizing participants’ socio-demographic characteristics and information related to domestic violence are presented in Table 1.

**Table 1:** Sociodemographic characteristics and domestic violence related information of the study participants (n = 250)

Characteristics	Frequency (%)
Age (years)	
(Mean ± SD)	32.0 (± 8.24)
Age range	18–49
Sex	
Male	94 (37.6%)
Female	156 (62.4%)
Marital status	
Married	249 (99.6%)
Separated	1 (0.4%)
Prevalence of Violence	
No	202(81.1%)
Yes	47 (18.9%)
Received Counselling about Violence at Home	
No	30 (63.8%)
Yes	17 (36.2%)

Table 2 presents the evaluation of the readiness and referral services for supporting women encountering Gender-Based Violence (GBV) across nine healthcare facilities. Among these, five were situated in rural or semi-urban areas, while four were in urban settings. The data reveal that merely 33.3% (n = 3) of facilities inquire about or identify cases of domestic violence. Additionally, only 11.1% (n = 1) of the assessed facilities provide referrals to non-governmental organizations (NGOs), counseling, or other external services. Moreover, merely 11.1% (n = 1) of healthcare providers received sexual violence response training within the preceding six months. Notably, no significant disparity was observed between baseline and endpoint assessments.

**Table 2:** Readiness of health facilities for services and referrals regarding GBV in Pakistan (n = 9)

Characteristics	Baseline (%)
Availability of the National guidelines for the provision of health care to women subjected to Domestic violence	
Yes	1 (11.1)
No	8 (88.9)
Availability of the National guidelines for the provision of health care to women subjected to Sexual violence	
Yes	1 (11.1)
No	8 (88.9)
The facility asks about or identifies the DV cases	
Yes	3 (33.3)
No	6 (66.7)
Provision of broader medical care related to GBV	
Yes	3 (33.3)
No	6 (66.7)
The facility offers psychological support/crisis counselling/first-line support	
Yes	2 (22.2)
No	7 (77.8)
The facility makes referrals to NGOs, counselling, or any other services outside the health facility	
Yes	1 (11.1)
No	8 (88.9)
Provision of clinical injury treatment related to GBV	
Yes	3 (33.3)
No	6 (66.7)
The facility makes referrals of sexual assault and partner violence to the following:	
**Police**	2 (22.2)
Protection Service	2 (22.2)
Shelter Services	2 (22.2)
**Women NGO**	2 (22.2)
Crisis Counselling Services	2 (22.2)
Livelihood Support	2 (22.2)
Legal Services	2 (22.2)
Availability of a referral directory, with names and contact details of organizations/services	
Yes	3 (33.3)
No	6 (66.6)
Healthcare providers received training on responding to sexual violence in the last six months	
Yes	1 (11.1)
No	8 (88.9)
Healthcare providers received training on responding to intimate partner/domestic violence in the last six months	
Yes	1 (11.1)
No	8 (88.9)

Table 3 illustrates that one, two, or at most three facilities possess the necessary commodities. Merely 11.1% (n = 1) of the evaluated facilities displayed visible posters addressing violence against women. Additionally, in 88.9% (n = 7) of facilities, the Health Management Information System (HMIS) lacks a mechanism to document incidents of sexual assault or intimate partner/domestic violence. Remarkably, no notable distinction was discerned between baseline and endpoint assessments.

**Table 3:** Availability of commodities regarding violence against women in health care facilities in Pakistan (n = 9)

Clear signs in the clinic regarding days and times of GBV service	
Yes	1 (11.1)
No	8 (88.9)
The counselling rooms are separate and available for a private and confidential consultation	
Yes	1 (11.1)
No	8 (88.9)
The examination rooms are separate and available for private and confidential consultation	
Yes	3 (33.3)
No	6 (66.7)
There are visible posters about violence against women	
Yes	1 (11.1)
No	8 (88.9)
Health Management Information System (HMIS) includes means to record Sexual Assault	
Yes	1 (11.1)
No	8 (88.9)
HMIS records Intimate Partner/Domestic Violence	
Yes	1 (11.1)
No	8 (88.9)
Facility’s records, registers, and forms are kept in a secure storage	
Yes	2 (22.2)
No	7 (77.8)
Take-home cards or information do not have any direct indication of the Survivor’s Abuse	
Yes	1 (11.1)
No	8 (88.9)

**Fig. 1:**
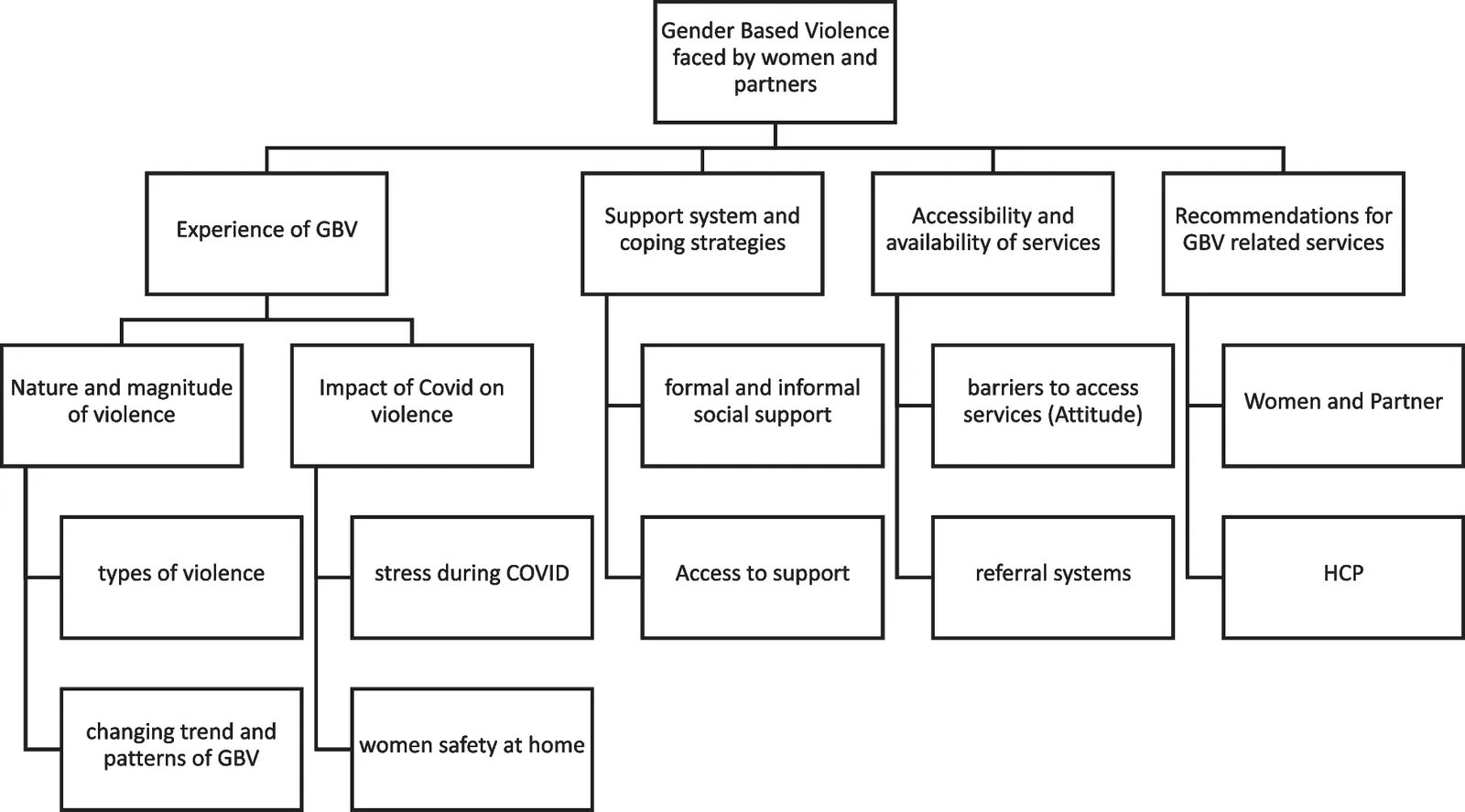
Qualitative thematic framework of gender-based violence experiences and service readiness (GBV = gender-based violence; HCP = healthcare provider)

A qualitative thematic framework was developed to demonstrate convergence and complementarity between gender- based violence experiences and service readiness (as shown in Fig. 1).

Interpreting findings through the WHO health system building blocks highlights gaps primarily at the service delivery, workforce capacity, and health information system levels, indicating structural weaknesses affecting GBV response during emergencies.

### Theme 1: experiences of GBV during COVID

This theme emerged from participants’ reflections on their experiences regarding the nature and extent of violence and the impact of COVID on violence.

### Nature and magnitude of violence

Violence against women escalated during COVID-19, with physical and sexual violence being predominant. Women frequently presented at clinics or emergency rooms with vaginal bleeding following instances of violence, often with the causes remaining unidentified or ambiguous. One healthcare provider shared a harrowing account: "A woman reported that her husband used to tie her hands and feet and then force her to have sex with him, without her agreement…. There were also cases of domestic violence, where abuse was observed during pregnancy."

Healthcare providers also noted an increase in violence cases during COVID, attributing it to heightened tensions at home due to increased time spent together, job loss, food scarcity, and financial constraints, resulting in quarrels and physical assaults. Women seldom reported assaults or sought medical help due to fear of family criticism and harassment.

Women also expressed feeling unsafe within their own homes. One woman recounted, "Violence has happened to me. My father and brother-in-law beat me for no reason and over trivial issues. They beat me, but Allah [God] gave me the strength to survive."

### Impact of COVID on violence

Families faced distress as job losses and business setbacks became commonplace. This led to heightened tensions within families, resulting in conflicts and violence. With social gatherings prohibited and individuals confined to their homes, feelings of loneliness and frustration escalated, contributing to a surge in violence. A woman shared her experience, saying, "During COVID, the entire family was at home. My husband had also lost his job, and we were in financial trouble. At home, there was a lot of stress and conflict, and my spouse used to beat my children and me."

One partner reflected on the impact of COVID on GBV, stating, "My job was not stable, and we were having financial problems. I lost control due to a variety of factors. My wife would not stop whining, so I yelled at her or hit her."

Many individuals faced unemployment, financial instability, and economic crises, which escalated disputes and, ultimately, violence. A woman who desired to work while managing household responsibilities faced disapproval from her family, compelling her to stay home and tend to domestic chores. As she expressed, "My mother-in-law is not very supportive. Even when I tried to convince her to let me look for work to earn money, I was stopped and told to focus on household work and kids."

### Theme 2: support system and coping strategies

This theme encompasses formal and informal social support and access to support.

### Formal and informal social support

During COVID, the availability of online treatment and counseling became crucial. As husbands’ mobility was restricted, frustration mounted, leading to violence against women. Hence, women sought advice on family planning and strategies to stay safe from violence, which could help alleviate conflicts, physical abuse, and mental anguish. NGOs like Ansar Burney and Edhi Foundation offer services, but societal stigma often deters women from seeking help. Families discourage their children from utilizing these services out of fear of shame and loss of societal dignity. One healthcare provider remarked, "Due to the prevailing cultural norms, acknowledging violence against women is challenging. It is often perceived as a norm within relationships, resulting in underreporting."

### Access to support

Women acknowledged that healthcare providers offered sexual and reproductive health (SRH) services and counseling, including family planning, but with limitations due to providers’ inadequate understanding of violence-related issues stemming from a lack of knowledge.

### Theme 3: accessibility and availability of services

This theme comprises barriers to accessing services and referral systems.

### Barriers to accessing services

COVID posed a significant obstacle to accessing services, but it did not impede violence-related services. Although clinicians identified several women as assault victims during assessments, these cases often went unreported. Despite the increase in violence during COVID-19, healthcare providers lacked specific training on managing such cases during the pandemic. However, they adhered to established protocols for reporting violence against expectant women to senior management.

### Referral systems

In Pakistani society, seeking counseling is discouraged and viewed negatively. Healthcare providers noted the absence of services specifically addressing violence against women at their facilities. Despite the surge in violence during COVID-19, the need for facilities to refer violence-affected women was keenly felt.

### Theme 4: recommendations for GBV-related services

Recommendations for GBV-related services are divided into recommendations for women and partners and recommendations for healthcare providers.

### Women and partners

During COVID, online treatment, counseling, and tips for maintaining family harmony and preventing violence are crucial. Emphasizing mutual understanding can effectively prevent physical and mental abuse, fostering a more respectful environment. Involving family and religious leaders (Ulemma) in addressing violence against women is crucial.

One male partner emphasized the role of governmental institutions in addressing issues, expressing awareness of Sindh governmental departments undertaking such work.

### Healthcare providers

The COVID pandemic underscores the need for healthcare providers to utilize technology for counseling services, if face-to-face interactions are not feasible, to prevent violence. Involving law enforcement, family members, and technology is vital in preventing violence in disputes. Documenting violent incidents can provide evidence against such behavior. A healthcare provider highlighted that their facility did not offer any services for GBV beyond examining rape cases and compiling reports for the police department, with no change observed during COVID-19.

## Discussion

This study elucidated the exacerbation of gender-based violence (GBV) during the COVID-19 pandemic, revealing significant deficiencies in Pakistan’s healthcare system regarding access to GBV services, guidelines, immediate care, psychosocial support, referrals, and documentation. These findings echo global reports that COVID-19 unleashed a *“shadow pandemic”* of violence against women under lockdown conditions, as women were isolated with abusers and support services were disrupted [4]. Early in the pandemic, UN agencies warned of sharp increases in GBV and projected millions of additional cases worldwide as preventative efforts were hampered by diverted resources and movement restrictions [13]. Empirical studies across diverse settings confirm reductions in GBV service provision and quality, increased provider workload, shifts to remote service utilization, and funding challenges during COVID-19 surges [14, 15]. For example, analyses from the United States and Argentina noted that domestic violence hotlines, shelters, and legal/health services faced strain or reduced capacity during lockdowns [14, 15]. These challenges persisted across both high-income and low- and middle-income countries, underlining critical gaps in disaster preparedness for GBV in health systems. In response, the World Health Organization (WHO) and other bodies urged health sectors to maintain essential GBV services during the pandemic – for instance, through telemedicine, hotlines, and proactive outreach as part of the COVID-19 health response [16]. Nurse-led and midwife-led initiatives have indeed shown promise as adaptive strategies; in Bangladesh, a pandemic-era telemedicine program led by midwives sustained maternal health services and incorporated GBV screening, effectively reaching women unable to attend clinics in person [17]. Such examples suggest that leveraging technology and task-sharing with nurses and midwives can help address GBV issues in LMICs even amid crises.

Although Pakistan passed the Domestic Violence (Prevention and Protection) Act in 2021, signifying formal condemnation of domestic violence and other forms of GBV, implementation remains weak at the individual, institutional, and societal levels [18]. While both formal mechanisms (e.g., laws, helplines, women protection centers) and informal community networks exist to address GBV, entrenched stigma and patriarchal norms often deter women from seeking help [6]. Pakistani women frequently do not report abuse or pursue institutional support due to fear of social shame, retaliation, or being disbelieved [6]. Cultural norms discouraging open discussion of family violence exacerbate this silence. In this context, survivors tend to rely on close family and friends for support, although those informal systems may lack the resources or knowledge to effectively intervene. Recognizing all forms of GBV as serious violations and enhancing social support are, therefore, paramount steps. The gap between legislation and practice highlights the need to not only enact laws but also to invest in enforcement, public awareness, and community engagement so that women feel safe to disclose abuse and access services.

Notably, this study’s novelty lies in examining GBV issues both during the acute pandemic and in the post-pandemic context, and from both the health system and survivor perspectives. By assessing healthcare system readiness alongside women’s experiences, the research provides a comprehensive view of the challenges and informs holistic recommendations. The dual focus on systemic capacity and on the barriers faced by victims extends understanding of GBV beyond the immediate COVID-19 crisis and into broader health system resilience. It underscores that the deficiencies observed are not merely a temporary byproduct of COVID-19 but reflect longstanding structural gaps that persist beyond emergencies. The recommendations emerging from this work address these gaps through multi-level strategies.

Importantly, the findings stress the need for developing long-term policies and guidelines that integrate GBV into maternal and reproductive health frameworks beyond pandemics and disasters. There is a consensus that sexual and reproductive health and rights (SRHR) initiatives must encompass GBV prevention and response as core components, rather than treating GBV as an isolated issue [10, 12]. Global health experts have called for comprehensive approaches that uphold women’s rights and safety in all settings, including during health emergencies [16, 19]. This implies institutionalizing routine GBV screening and referral in healthcare, ensuring that national emergency preparedness plans include GBV response, and strengthening coordination between health, social services, and law enforcement. Innovative, evidence-based approaches are needed to tackle GBV collectively and strategically. For example, digital health interventions (tele-counseling, SMS alert systems, mobile apps) and community-driven solutions (engaging local leaders, empowering women’s groups) have emerged as promising avenues to support survivors when mobility is limited [19, 20]. Recent research shows that well-designed GBV prevention programs can be effective and cost-efficient in LMICs, providing a strong rationale for sustained investment in scaling up these interventions [21]. Overall, strengthening health system readiness for GBV through provider training, resource allocation, privacy protections, data systems for GBV case tracking, and cross-sector collaborations is essential. Such measures would enable healthcare institutions to offer a supportive environment for GBV survivors at all times, and especially during future crises when vulnerabilities are heightened. In addition, gender-inclusive awareness campaigns targeting both men and women are essential to address harmful social norms, promote early help-seeking behavior, and strengthen community-level prevention of gender-based violence.

### Strengths and limitations

A key strength of this study is its mixed-methods design, which enabled triangulation between quantitative facility assessments and qualitative perspectives from women, partners, and healthcare providers. This comprehensive approach provided a nuanced understanding of system readiness and survivor experiences during an unprecedented global crisis. Additionally, data collection across multiple facility levels (primary to tertiary) enhanced contextual validity. However, limitations include the study’s focus on a single province, which may limit generalizability, and potential underreporting due to the sensitivity of GBV topics and prevailing sociocultural taboos. Because the study primarily recruited clients attending reproductive health services, the majority were married individuals; therefore, findings may not capture GBV experienced among unmarried populations or violence perpetrated by extended family members. Future research should expand geographically and incorporate participatory approaches involving survivors and frontline providers to deepen insights into effective intervention strategies.

## Conclusion

This study identifies significant gaps in healthcare facilities’ readiness to address violence against women, including deficiencies in national norms, resources, identification procedures, referral services, and privacy measures. Healthcare providers’ inadequate training may hinder their ability to adequately support GBV victims. Urgent actions are needed, including the development of guidelines, provision of resources, implementation of training programs, improvement of identification procedures, expansion of referral networks, strengthening of privacy safeguards, and enhancement of awareness campaigns. These measures are essential to foster a supportive environment in healthcare institutions for GBV victims.

## Supplementary Information

Supplementary Material 1.

## Data Availability

Access to the data used in this analysis may be obtained upon request from the authors.
