# Supplementary material for: Healthcare systems’ readiness to address gender-based violence during COVID-19 and beyond in Pakistan: a mixed methods study
Source: Reprod Health. 2026 Aug 19;22(Suppl 3):281. doi: 10.1186/s12978-026-02356-3 (PMC13487947; doi:10.1186/s12978-026-02356-3)
Supplement: Supplementary file 1 [file 12978_2026_2356_MOESM1_ESM.docx]

**Supplementary File S 1**

**Joint Display Integrating Quantitative and Qualitative Findings**

**Supplementary Table S1. Integration of Facility Readiness Indicators and Qualitative Themes**

| **WHO Readiness Domain** | **Quantitative Findings** | **Supporting Qualitative Themes** | **Interpretation** |
| --- | --- | --- | --- |
| Service delivery | Only 33.3% facilities screened for domestic violence; 22.2% offered psychological support | Women reported limited identification of violence and lack of dedicated services | Screening and response services remain inconsistently implemented |
| Health workforce | 11.1% providers trained in GBV response | Providers expressed lack of training and uncertainty managing GBV cases | Workforce capacity gaps significantly limit GBV response |
| Health information systems | HMIS documented GBV in only 11.1% facilities | Providers reported absence of reporting tools and documentation systems | Weak information systems contribute to underreporting |
| Referral systems | 11.1% facilities had referral linkages | Participants reported difficulty accessing external services and stigma barriers | Referral networks are fragmented and poorly institutionalized |
| Infrastructure / privacy | Only 33.3% facilities had private examination rooms | Women expressed concerns about confidentiality and safety | Infrastructure limitations discourage disclosure |
| Governance / policy | National GBV service guidelines available in 11.1% facilities | Providers reported unclear institutional protocols | Governance gaps hinder standardized response |

**Supplementary File S2**

**COREQ (32-Item) Checklist**

| **Domain** | **No.** | **Item** | **Information reported in manuscript** | **Section** |
| --- | --- | --- | --- | --- |
| Research team and reflexivity | 1 | Interviewer/facilitator | Interviews were conducted by trained male and female research data collectors. | Methods – Data collection |
|  | 2 | Credentials | Research team included PhD-level investigators and trained research staff. | Author list / Methods |
|  | 3 | Occupation | Interviewers were trained research staff working within the study team. | Methods |
|  | 4 | Gender | Female researchers interviewed women and male researchers interviewed men. | Methods – Data collection |
|  | 5 | Experience and training | Data collectors were trained in qualitative interviewing procedures before data collection. | Methods |
|  | 6 | Relationship established | No prior relationship was established with participants before recruitment. | Methods |
|  | 7 | Participant knowledge of interviewer | Participants were informed about study objectives and interviewer roles during consent. | Methods – Ethics |
|  | 8 | Interviewer characteristics | Interviews followed standardized guides to minimize interviewer bias. | Methods |
| Study design | 9 | Methodological orientation | Qualitative data were analyzed using content analysis. | Methods – Data analysis |
|  | 10 | Sampling | Purposive sampling was used for qualitative interviews until saturation. | Methods – Sampling |
|  | 11 | Method of approach | Participants were approached at participating health facilities during service visits. | Methods – Recruitment |
|  | 12 | Sample size | Qualitative participants included women, partners, and 33 healthcare providers. | Methods – Participants |
|  | 13 | Non-participation | Participants unwilling to participate were excluded after refusal of consent. | Results – Participants |
|  | 14 | Setting of data collection | Interviews were conducted in separate rooms within healthcare facilities. | Methods – Data collection |
|  | 15 | Presence of non-participants | Interviews were conducted privately without the presence of non-participants. | Methods |
|  | 16 | Description of sample | Participant demographic characteristics are presented in Table 1. | Results – Table 1 |
|  | 17 | Interview guide | Semi-structured interview guides were developed, reviewed by experts, and pilot tested. | Methods – Instruments |
|  | 18 | Repeat interviews | Repeat interviews were not conducted. | Methods |
|  | 19 | Audio/visual recording | Interviews were audio recorded with participant consent. | Methods – Data collection |
|  | 20 | Field notes | Interviewers documented nonverbal cues and observational notes. | Methods |
|  | 21 | Duration | Interviews lasted approximately 40–60 minutes. | Methods |
|  | 22 | Data saturation | Interviews continued until thematic saturation was achieved. | Methods – Sampling |
|  | 23 | Transcripts returned | Transcripts were not returned to participants for checking. | Methods |
| Analysis and findings | 24 | Number of data coders | Multiple researchers were involved in coding and reviewing transcripts. | Methods – Data analysis |
|  | 25 | Description of coding tree | Data were coded into categories, subcategories, and themes through content analysis. | Methods |
|  | 26 | Derivation of themes | Themes were derived inductively from interview data. | Methods |
|  | 27 | Software | Statistical analysis used SPSS; qualitative coding conducted manually by research team. | Methods |
|  | 28 | Participant checking | Participant feedback on findings was not conducted. | Methods |
|  | 29 | Quotations presented | Participant quotations are included in qualitative results sections. | Results |
|  | 30 | Data and findings consistent | Findings are supported by quantitative tables and qualitative quotations. | Results |
|  | 31 | Clarity of major themes | Major qualitative themes are presented in Figure 1 and results narrative. | Results |
|  | 32 | Clarity of minor themes | Subthemes and supporting categories are described within qualitative results. | Results |
